# Effects of E-Cigarette Liquid Ratios on the Gravimetric Filter Correction Factors and Real-Time Measurements

**DOI:** 10.4209/aaqr.230011

**Published:** 2023-07-28

**Authors:** Austin Close, Jane Blackerby, Heather Tunnell, Jack Pender, Eric Soule, Sinan Sousan

**Affiliations:** 1 Department of Public Health, Brody School of Medicine, East Carolina University, Greenville, NC 27834, USA; 2 Department of Chemistry, East Carolina University, Greenville, NC 27858, USA; 3 Department of Health Education and Promotion, College of Health and Human Performance, East Carolina University, Greenville, NC 27858; 4 North Carolina Agromedicine Institute, Greenville, NC 27834, USA

**Keywords:** Particulate matter, PM_2.5_, ECIG, PG/VG ratios, Filter correction factors

## Abstract

Electronic cigarettes (ECIGs) generate high concentrations of particulate matter (PM), impacting the air quality inhaled by humans through secondhand exposure. ECIG liquids are available commercially and some users create their own “do-it-yourself” liquids, and these liquids often vary in the amounts of their chemical ingredients, including propylene glycol (PG) and vegetable glycerin (VG). Previous studies have quantified PM concentrations in ECIG aerosol generated from liquids containing different PG/VG ratios. However, the effects of these ratios on aerosol instrument filter correction factors needed to measure PM concentrations accurately have not been assessed. Thus, ECIG aerosol filter correction factors for multiple aerosol instruments (SMPS + APS, MiniWRAS, pDR, and SidePak) were determined for five different PG/VG ratios 1) 0PG/100VG, 2) 15PG/85VG, 3) 50PG/50VG, 4) 72PG/28VG, and 5) 90PG/10VG and two different PM sizes, PM_1_ (1 μm and smaller) and PM_2.5_ (2.5 μm and smaller). ECIG aerosols were generated inside a controlled exposure chamber using a diaphragm pump and a refillable ECIG device for all the ratios. In addition, the aerosol size distribution and mass median diameter were measured for all five ECIG ratios. PM_2.5_ correction factors (5–7.6) for ratios 1, 2, 3, and 4 were similar for the SMPS + APS combined data, and ratios 1, 2, 3 were similar for the MiniWRAS (~2), pDR (~0.5), and SidePak (~0.24). These data suggest different correction factors may need to be developed for aerosol generated from ECIGs with high PG content. The higher correction factor values for the 90PG/10VG ratio may have resulted from greater PG volatility relative to VG and sensor losses. The correction factors (ratios 1–4) for PM_2.5_ were SMPS + APS data (4.96–7.62), MiniWRAS (2.02–3.64), pDR (0.50–1.07), and SidePak (0.22–0.40). These data can help improve ECIG aerosol measurement accuracy for different ECIG mixture ratios.

## 1 INTRODUCTION

Electronic cigarettes (ECIGs) are devices that aerosolize a liquid for user inhalation (Breland *et al*., 2017). ECIG liquid contains a mixture of propylene glycol (PG), vegetable glycerin (VG), nicotine, and added flavoring chemicals (Grana *et al*., 2014; Sousan *et al*., 2022b). ECIG devices utilize an atomizer to produce the airborne contaminants containing particulate matter (PM) and volatile organic compounds (VOCs) (Fernández *et al*., 2015; Tzortzi *et al*., 2018; Marcham and Springston, 2019). Much of the current laboratory research on ECIGs is conducted using an airborne exposure chamber, which allows the measurement of aerosol emissions generated from ECIGs in a controlled environment (Czogala *et al*., 2014; Geiss *et al*., 2015; Cunningham *et al*., 2020; Soule *et al*., 2022).

While the long-term health effects of ECIG use are still unknown, ECIG use is still associated with negative health outcomes and represents a public health challenge (Walley *et al*., 2019). The World Health Organization (WHO) reports that over 1.1 billion adults use electronic nicotine delivery systems like ECIGs (WHO, 2020). In the United States, daily ECIG use has trended upward in recent years, particularly among younger adults (Obisesan *et al*., 2020), and 50% of adolescents experience secondhand exposure to cigarette aerosol or ECIG aerosol (Gentzke *et al*., 2019). In addition, VOCs generated from ECIGs, such as formaldehyde, acetaldehyde, acrolein, nitrosamines, chloropropanols and furans, have been associated with negative health outcomes (Vardavas *et al*., 2012; Soussy *et al*., 2016; Ogunwale *et al*., 2017; Lestari *et al*., 2018).

Some of the PM generated by ECIG use is PM_2.5_ (PM 2.5 μm and smaller in diameter), or fine PM, which poses an even greater risk to the human respiratory system due to their ability to infiltrate deeper portions of the lungs where air exchanges with blood circulating throughout the body (Choi *et al*., 2010). The rapid transport of PM_2.5_ to blood circulating in the body could pose severe health risks, as an increase in PM_2.5_ air pollutants has been associated with a higher risk of cardiac arrhythmias (Johnson, 2004). In addition, PM_2.5_ deposits on the respiratory bronchioles when inhaled, and ultrafine particles, defined as PM_1_ (PM 1 μm and smaller in diameter), are included in PM_2.5_ and can deposit on the alveoli when inhaled (Johnson, 2004). These ultrafine particles pose the greatest risk for ischemic heart disease (Schulz *et al*., 2005; Schraufnagel, 2020). The myriad of negative health outcomes associated with exposure to aerosols generated from ECIGs indicates the need to measure PM concentrations in indoor settings.

Gravimetric filter analysis is considered the gold standard of aerosol measurements and is an optimal method for measuring the true PM used in laboratory or field research (Halterman *et al*., 2018; Olegario *et al*., 2021). This analysis is achieved by weighing differences in mass concentrations for a specific flow rate and sampling time and can be used to measure ECIG-generated PM concentrations (Sousan *et al*., 2021). Despite its accurate assessment of aerosol, gravimetric analysis can only capture discrete (average) concentrations over the sampling period (Sousan *et al*., 2022a). Real-time monitors like optical particle counters use the magnitude of light scattered from particles to calculate the mass concentration of different PM sizes and can provide the temporal information that gravimetric analysis lacks (van Drooge *et al*., 2019; Sousan *et al*., 2021, 2022a). However, real-time optical monitors are calibrated differently by the manufacturers and are either calibrated using a specific aerosol source such as dust or calibrated with different polystyrene latex particles at specific sizes (Sousan *et al*., 2016a, 2016b). Due to these calibration differences, the optical monitor could overestimate or underestimate the true aerosol mass concentration while measuring a different aerosol source from calibration. In addition, due to different calibration methods acquired by manufacturers, the optical monitors could report different concentrations while measuring the same exposure side-by-side. Therefore, when utilizing these light-scattering devices, manufacturers highly recommend developing an on-site filter correction factor or an aerosol source-specific filter correction factor for the optical monitor (GRIMM, 2021). The correction factor for an optical monitor is developed by collocating the optical monitor with a discrete filter for gravimetric analysis over a specific time period (Olegario *et al*., 2021). Then, the optical device-specific filter correction factor is calculated by dividing the discrete filter measurement by the average real-time measurement acquired by the optical monitor. The real-time measurements for the optical monitor are then corrected by multiplying the filter correction factor by the frequency of measurements acquired by the optical monitor. Therefore, the filter correction factor pushes the real-time measurements toward the true concentrations. Sousan *et al*. (2022a, 2023) measured the gravimetric analysis for several ECIGs and optical aerosol monitors and determined that the filter correction factors are specific to different aerosol monitors and ECIG brands.

Several laboratory studies have measured PM_2.5_ concentrations in ECIGs and evaluated the effects of different PG/VG ratios. Li *et al*. (2020) measured the effect of PG/VG ratios in conjunction with 2.4% nicotine and the absence of nicotine. The study reported that higher PG content in the ECIG liquid composition containing nicotine significantly decreased PM_2.5_ concentrations due to the high volatility of PG. PG/VG ratio may also impact user experience, where higher VG content provides a larger cloud compared to higher PG content (Harvanko *et al*., 2019). Ooi *et al*. (2019) aerosolized four PG/VG ratios (100/0, 20/80, 50/50, and 80/20) using an Innokin E-cig device with a flow rate of 2 L min^−1^ and a puffing profile of 3 seconds. The authors found that aerosol generation and toxins produced were higher with larger amounts of VG, due to the lower volatility of VG. In addition, Lampos *et al*. (2019) showed that PM aerosolized from a 20PG/80VG liquid solution provided higher concentrations compared to a solution that was 50PG/50VG. Therefore, the effects of different PG/VG ratios on aerosol generation have been established, but the effect of PG/VG ratios on the filter correction factors derived for different aerosol instruments has not been established, indicating a need for this study.

The objective of the current study was to determine filter correction factor values at different PG/VG ratios for different aerosol monitors by using an ECIG device to aerosolize liquids containing 5% nicotine. The study will show if different PG/VG ratios affect the filter correction factor calculated for each aerosol monitor. Filter correction factor values were calculated for different particle sizes (PM_1_ and PM_2.5_) and five different PG/VG ratios across a consistent ECIG brand by using gravimetric analysis and real-time monitors. In addition, the aerosol size distributions of the different ratios were measured.

## 2 MATERIALS AND METHODS

The focus of the study was measuring the PG/VG ratios for two particle sizes, PM_1_ and PM_2.5_, since the Sousan *et al*. (2022a) study showed that the ECIG particles are mainly 2.5 μm and smaller.

### 2.1 Real-Time Aerosol Monitors

#### 2.1.1 MiniWRAS

The GRIMM Mini Wide-Range Aerosol Spectrometer (MiniWRAS 1371, Grimm Aerosol Technik, Ainring, Germany) was chosen due to capability of measuring PM ranging between 0.01 μm and 35 μm in 41 bins. The defining capability of the MiniWRAS that makes it conducive to this study is the ability to measure PM_1.0_ and PM_2.5_ in real time. The MiniWRAS is the only device that can simultaneously measure micron-sized and nanoparticles. The device measures larger particles (> 0.25 μm) and smaller particles (< 0.25 μm) using an optical sensor and a corona charger, respectively. The MiniWRAS measures number concentration and then converts to mass concentration between 0 and 100,000 μg m^−3^ in real-time. The device runs at a flow rate of 1.2 L min^−1^, and data is collected on the MiniWRAS at a one-minute interval.

#### 2.1.2 pDR 1500

The personal DataRAM (pDR 1500, Thermo Scientific, Franklin, MA, USA) is a real-time photometer which utilizes a cyclone at a pre-determined flow rate to detect PM at specific sizes. The device was chosen because it is equipped with a 37-mm filter holder that is an integral component of the monitor, as it can accurately detect PM using gravimetric analysis. The pDR can measure PM concentrations between 0 and 400,000 μg m^−3^ in real-time. Two different pDRs were utilized. One was used to measure PM_1_ and the other to measure PM_2.5_ filter and real-time data. Each pDR operated at a specific flow rate and was equipped with pertinent cyclones to measure PM_1_ and PM_2.5_ at flow rates of 3.46 and 1.52 L min^−1^, respectively. Real-time data were collected by each pDR with a 1-second frequency.

#### 2.1.3 SidePak

The SidePak personal aerosol monitor (AM520, TSI Inc., Shoreview, MN, USA) is a real-time photometer which is capable of measuring PM at precise sizes using different impactors. The SidePak AM510 (previous model) has been frequently used in studies by the ECIG research community, mainly due to its small size and relatively low cost compared to other reference instruments (Lee *et al*., 2017; Melstrom *et al*., 2017; Soule *et al*., 2017; Chen *et al*., 2018). This, coupled with its ability to measure specific PM using an internal impactor, makes this device apt for this study. The device measures aerosols at mass concentrations between 0 and 100,000 μg m^−3^. Sampling using the SidePak occurred with a PM_2.5_ impactor and a flow rate of 1.8 L min^−1^ with a 1-second sampling frequency.

#### 2.1.4 SMPS

The Scanning Mobility Particle Size Spectrometer (SMPS 3938, TSI, Shoreview, Minnesota, USA) is a real-time instrument that can measure the size of particles using 191 bins and measures particles from 0.001 μm to 1.0 μm (Sousan *et al*., 2017, 2018). The SMPS was chosen because it is considered the laboratory gold-standard for real-time measurements of particles smaller than 1 μm. The SMPS contains the 3081A Long Differential Mobility Analyzer, a 3756 Ultrafine Condensation Particle Counter, and an aerosol X-ray neutralizer. Therefore, the SMPS uses differential mobility to separate particle sizes that are then counted by the particle counter (Wang and Flagan, 1990). The SMPS measures particle number concentration in a range of up to 10^7^ particles cm^−3^, then converts to mass concentration assuming a density of the measured aerosol. The SMPS was operated with a 0.3 L min^−1^ aerosol flow rate and a 2.0 L min^−1^ sheath flow rate and with a 1-min sampling frequency.

#### 2.1.5 APS

An Aerodynamic Particle Sizer (APS 3321, TSI, Shoreview, Minnesota, USA) was utilized due to its ability to measure broad particle size ranges larger than the SMPS (Sousan *et al*., 2016a, 2016b). Therefore, the APS was chosen to complement the SMPS for particles larger than 1 μm. The APS is a spectrometer that uses Time-Of-Flight it takes for a particle to travel between two finite distances to measure the particle size that is then counted by the device (Baron, 1986). The APS can measure particle size from 0.5–20 μm in 52 channels and a maximum particle number concentration of up to 10,000 particles cm^−3^, then converts to mass concentrations assuming density of the measured aerosol. The APS operated at a flow rate of 5 L min^−1^ and was set to measure aerosol at a 1-min sampling frequency.

### 2.2 Gravimetric Analysis

The pDRs were equipped with 37-mm fiberglass filters (Whatman, CAT Non.1827–037, Maidstone, United Kingdom), and gravimetric analysis was conducted with the measurements. A Mettler Toledo microbalance (Microbalance XPR26DR, Mettler Toledo, Ohio, USA) and an anti-static kit with a large U-electrode (Model: 63052302, Mettler Toledo, Columbus, Ohio, USA) were utilized to measure pre- and post-weight filter measurements to determine the mass concentration. The difference between weights with sampling time and the flow rate was used to calculate the mass concentration. Filter equilibration for temperature and humidity were set for 22 ± 1°C and 45 ± 5%, respectively (Hinds, 1999).

### 2.3 Experimental Setup

#### 2.3.1 Chamber description

**Fig. 1.**
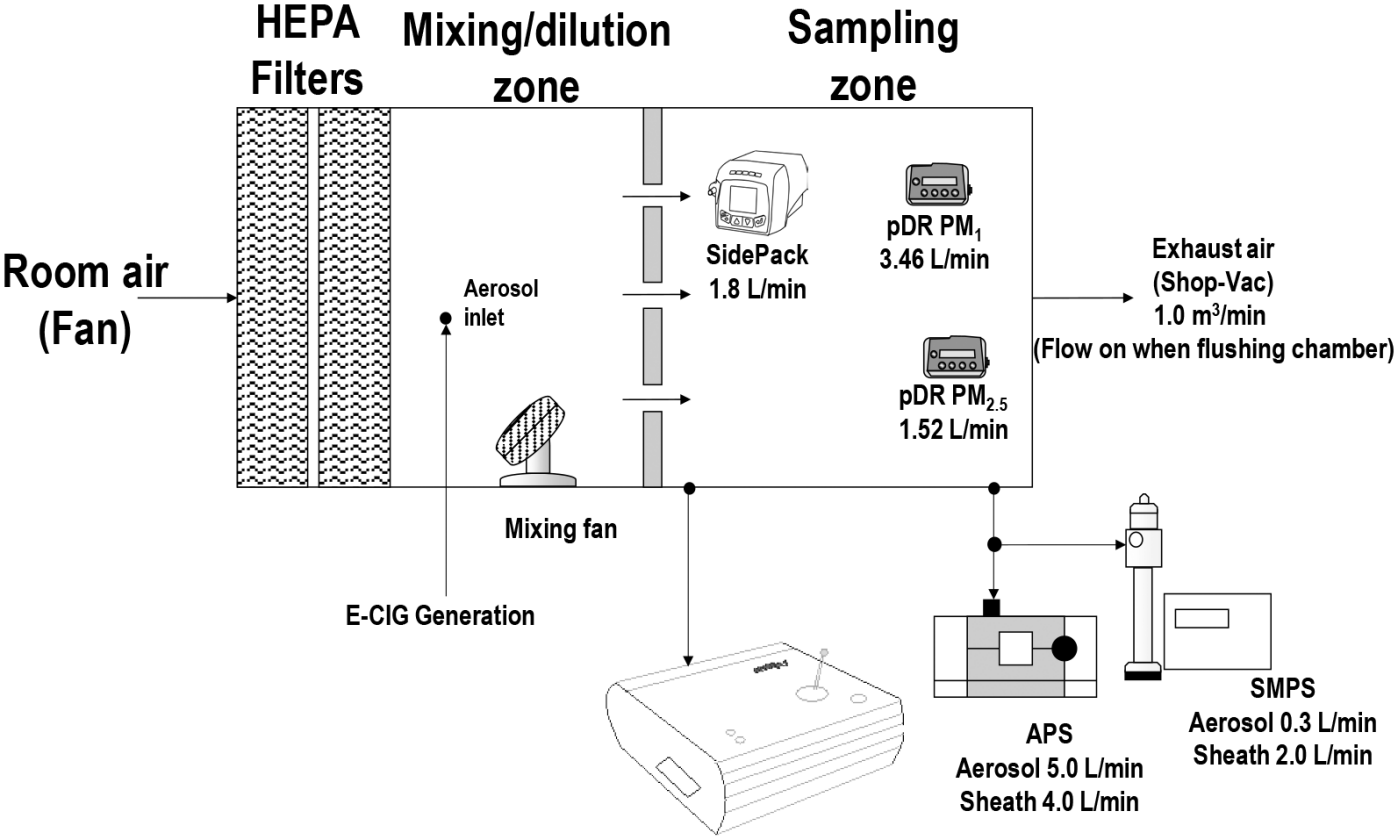
The experimental chamber for measuring ECIG exposure of different mixture ratios.

Experiments were performed inside a 0.5 m^3^ exposure chamber for the controlled aerosol generation and subsequent assessment, as shown in Fig. 1. The exposure chamber contained two halves: one half operated as the mixing zone (0.25 m^3^), and the second half operated as the sampling zone (0.25 m^3^). Two 99.99% efficiency HEPA filters were used for flushing the chamber with particle-free air at a rate of 1.0 m^3^ min^−1^. Air was removed from the chamber using a vacuum equipped with HEPA filters and a carbon filter. The two zones were dichotomized using a honeycomb (AS100, Ruskin, Grandview, MO, USA) flow straightening section, which permitted an even distribution of air in the sampling zone at a flow rate of 0.20 m^3^ min^−1^ from the two fans inside the mixing zone. The two fans mixed the air in the generation zone during the experiment and when the chamber was flushed post-experiment. The pDR and SidePak were placed inside the sampling zone. The SMPS, APS, and MiniWRAS were placed outside the chamber while sampling directly from the sampling zone. In previous work related to ECIG aerosol generation (Sousan *et al*., 2022a), the aerosol homogeneity of the sampling zone was ~9% tested by positioning optical sensors in the corners and the middle of the sampling zone The chamber temperature was 22 ± 2°C, and relative humidity was set at 45 ± 5%.

#### 2.3.2 PG and VG liquid mixture preparation

PG (MP Biomedicals, PubChem CID 1030) and VG (Thermo Scientific, 99% Glycerol, PubChem CID 753, Solon, OH, USA) liquids were used to prepare five ECIG mixtures. Liquids were prepared targeting PG/VG volume ratios of 0:100, 25:75, 50:50, 75:25, and 90:10, each containing 5% nicotine by weight (Thermo Scientific, 99% Nicotine, PubChem CID 89594, Franklin, MA, USA), and their respective density values were calculated as shown in Table 1. The density of PG (1040 mg mL^−1^) and VG (1260 mg mL^−1^) were multiplied by the volume ratio and summed to calculate the density of each ratio. A Hotplate (Cimarec+, Thermo Scientific, Waltham, MA) was used to heat and stir each of the mixtures, and the device was set at a temperature of 120°C and 300 revolutions per minute. A SMOK Novo 2 (SMOKTech, 2022) ECIG device was chosen to aerosolize the liquid mixtures. In addition, a PG/VG ratio of 100/0 was tested, and no aerosol concentrations were detected by the aerosol instruments and filters in the sampling zone. The 100% PG liquid aerosol was slightly visible at the entrance of the chamber in the mixing zone. Still, due to the volatility of PG and the mixing fans, the particles were most likely volatilized before entering the sampling zone. Therefore, a ratio of 90/10 was chosen as a substitute. This was likely due to the high volatility of PG in the mixing zone and the elimination of PG from the particle phase completely into the gas phase (David *et al*., 2020; Luo *et al*., 2021). After the experiments, the liquids were chemically analyzed, as shown in Table 1, to confirm the prepared PG/VG ratios, except for the 50PG/50VG ratio due to the lack of sufficient liquid. The chemical analysis is explained in detail in the Supplemental Information document. The chemical analysis indicated that the 25PG/75VG ratio was 15/85, the 75PG/25VG ratio was 72/28, and the 90PG/10VG ratio was 90/10. The lower PG values in the chemical analysis could have occurred due to PG volatilization during the mixing and heating process. Therefore, mixtures with chemical analysis will be used throughout this work. This device uses refillable pods and for each PG/VG ratio, a single pod was used to ensure no contamination of liquids with differing PG/VG ratios.

#### 2.3.3 Aerosol generation

A diaphragm pump (Thomas 1420–0504, Gardner Denver, Davidson, NC) and a clock generator (TFIS 12–240VUC 1CO CG, Weidmüller Interface GmbH & Co. KG, Detmold, Germany) were utilized to generate aerosol with the SMOK Novo 2. The clock generator controlled the ECIG puffing time, and 3-second puffs with 30-second intervals (3 seconds ON and 27 seconds OFF) with a flow rate of 2.0 L min^−1^ were performed which is representative of ECIG user puffing topography (Hiler *et al*., 2020). The chamber input air and vacuum air were turned off during the experiment to prevent aerosol dilution during the experiment. The experiments were repeated three times with each PG/VG ratio using three new empty pods purchased before the experiments and filled with each ratio, yielding 15 total experiments. Pods were filled and changed for every experiment and the ECIG device was fully charged at the beginning of each experiment.

### 2.4 Data Analysis

#### 2.4.1 Real-time mass concentrations

The concentrations of particulate matter measured from the pDRs and the SidePak were averaged over 1 minute and paired with the MiniWRAS data, which was collected for the same duration. The SMPS and APS data were combined using MATLAB (R2022a) code that calculated PM_1_ and PM_2.5_ for each ratio using the respective density values listed in Table 1. These concentrations were plotted on Box and Whisker plots for all the aerosol monitors. Concentrations were measured by particle size at PM_1_ and PM_2.5_ for the SMPS + APS, MiniWRAS, and pDRs. The SidePak measured particle size at PM_2.5_.

#### 2.4.2 Filter correction factors

Discrete filter mass concentrations were determined for each particle size and ECIG liquid ratio and divided by the average real-time mass concentration measured by the PM monitors to calculate the correction factor for the respective monitor and particle size.

The filter correction factor for each optical monitor was calculated using the following equation:

(1) $$\text{Device Specific Filter Correction Factor}=\frac{\text{Discrete Filter Mass Concentration}}{\text{Average Real-time Measurment of the Device}}$$

The filter correction factors indicate discrepancies between the real-time PM concentrations and the discrete PM concentrations measured by filters. The averages and standard deviations for each ratio were calculated for the three trials. Finally, the filter correction factors were plotted for each aerosol monitor, size, and ratio.

#### 2.4.3 Aerosol size distribution

The aerosol size distribution by mass for the generated aerosol for each ratio was measured with the MiniWRAS and combined SMPS and APS data. The SMPS and APS data were combined using a curve-fitting lognormal distribution function with TSI’s Data Merge Software (Model 390069 TSI, Shoreview, Minnesota, USA). The SMPS + APS curve was fitted using the “Use Fit Estimate Method”, which estimated the total for the overlapping region between the two and then used these two estimated totals. The mass median diameter (MMD) and geometric standard deviation (GSD) were also calculated using the same methods for the MiniWRAS and SMPS and APS data. For each of the 5 ratios, the aerosol particle size distribution for the MiniWRAS and SMPS + APS fitted data were plotted. The densities for each ratio differed based on their composition, and each corresponding density ratio was used to calculate the appropriate particle size distribution, MMD and GSD. The shape factor of each ratio was assumed 1.0 for the liquid aerosol (Hinds, 1999; Sousan *et al*., 2021).

## 3 RESULTS AND DISCUSSION

### 3.1 Real-Time Mass Concentrations

**Fig. 2.**
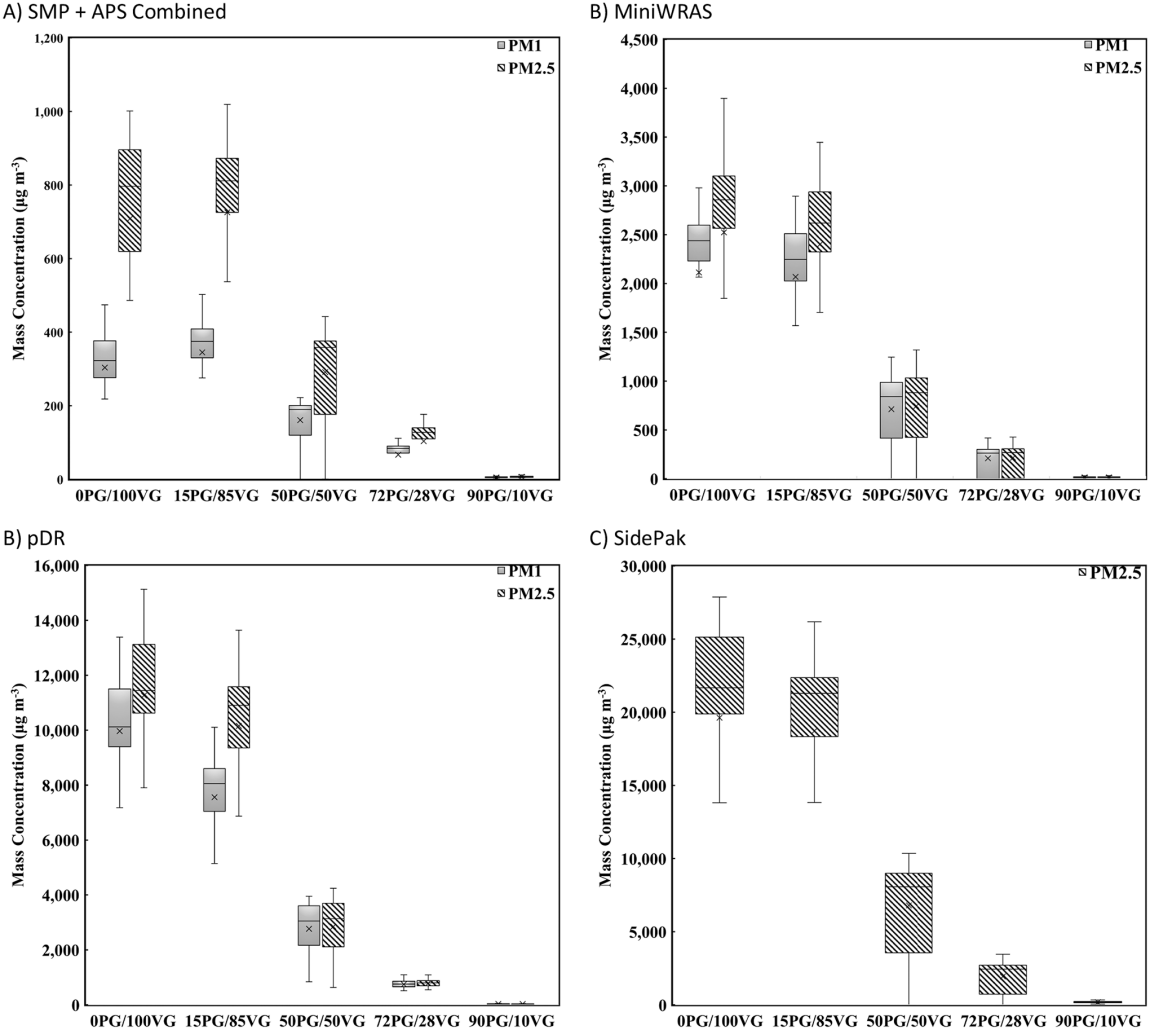
Box-Whisker plots for (A) SMPS + APS Fitted Data, (B) MiniWRAS, (C) pDR, and (D) SidePak. The SMPS + APS, MiniWRAS, and two pDRs measured PM_1_ and PM_2.5_. The SidePak measured PM_2.5_. The figures show measurements that represent the non-filter corrected data for the 5 ratios.

The real-time raw measurements for the SMPS + APS, MiniWRAS, pDR, and SidePak are shown in Fig. 2. In addition, the average PM_1_ and PM_2.5_ concentrations (μg m^−3^) of each ratio for the SMPS + APS, MiniWRAS, pDR, and SidePak are shown in Table S1 in the Supplemental Information. Aerosol concentrations varied across PM monitors for each ECIG ratio and particle size. The concentrations were lowest for liquids with the highest concentrations of PG. In addition, for the MiniWRAS and pDR, the PM_1_ and PM_2.5_ values become similar when PG values increase to 50% and higher indicating that the majority of particles are smaller than 1 μm. This could be due to the volatilization of particles larger than 1 μm at higher PG ratios or the particles generated are 1 μm and smaller.

Differences in PM_1_ and PM_2.5_ mass concentrations between aerosol monitors are attributed to the different sciences used to measure aerosol mass concentrations (Sousan *et al*., 2016a, 2016b, 2022a). Instruments based on optics are usually less accurate than monitors based on differential mobility or Time-Of-Flight measurements. Therefore, it is not surprising to see different values between these devices, given the different calibration methods between the optical sensors and the different sciences between the optical sensors and the SMPS and APS monitors. Previous work performed by Sousan *et al*. (2022a) with ECIG aerosol measurements also showed that the optical sensors provided different values among the optical monitors and the SMPS monitor.

The 0PG/100VG ratio generated the highest concentrations for all PM sizes, while 90PG/10VG ratio had the lowest concentrations across all PM sizes. The vast difference was likely caused by the volatility with high amounts of PG and smaller amounts of VG. The larger quantity of VG, compared to PG, caused a decrease in volatility in ECIG liquid, likely caused by the substantially lower vapor pressure in VG (Lampos *et al*., 2019; Sousan *et al*., 2022b). These results are consistent with previous research that has shown that ECIG liquids with a higher concentration of VG, have higher concentrations of aerosol generated due to the high volatility of PG and the low volatility of VG (David *et al*., 2020; Luo *et al*., 2021). In addition, Li *et al*. (2021) generated ECIG aerosol using 5 liquid PG/VG ratios (100:0, 70:30, 50:50, 30:70, 0:100) and found that the concentration decreases when PG values increase.

The SidePak reported the highest concentrations (up to 25,000 μg m^−3^) amongst the PM monitors, followed by the pDR (up to 13,125 μg m^−3^) and the MiniWRAS (up to 3,100 μg m^−3^), and the SMPS + APS data (up to 900 μg m^−3^) reported the lowest concentrations. These results are similar to Sousan *et al*. (2022a), where ECIG aerosol was generated in the same chamber and under similar settings for the VooPoo Drag 2, JUUL, NJOY Ace, NJOY Daily, and Hyde ECIG brands. The authors exhibited similar results, where the SidePak measured the highest concentrations, followed by the pDR and the MiniWRAS. The SidePak is known to overestimate the ECIG concentrations up to 80%, compared to the pDR, which overestimates by 50%, and the MiniWRAS, which underestimates by a factor of 2 or higher depending on the ECIG brand (Sousan *et al*., 2022a). However, this study did not control for PG/VG ratio.

### 3.2 Filter Correction Factors

**Fig. 3.**
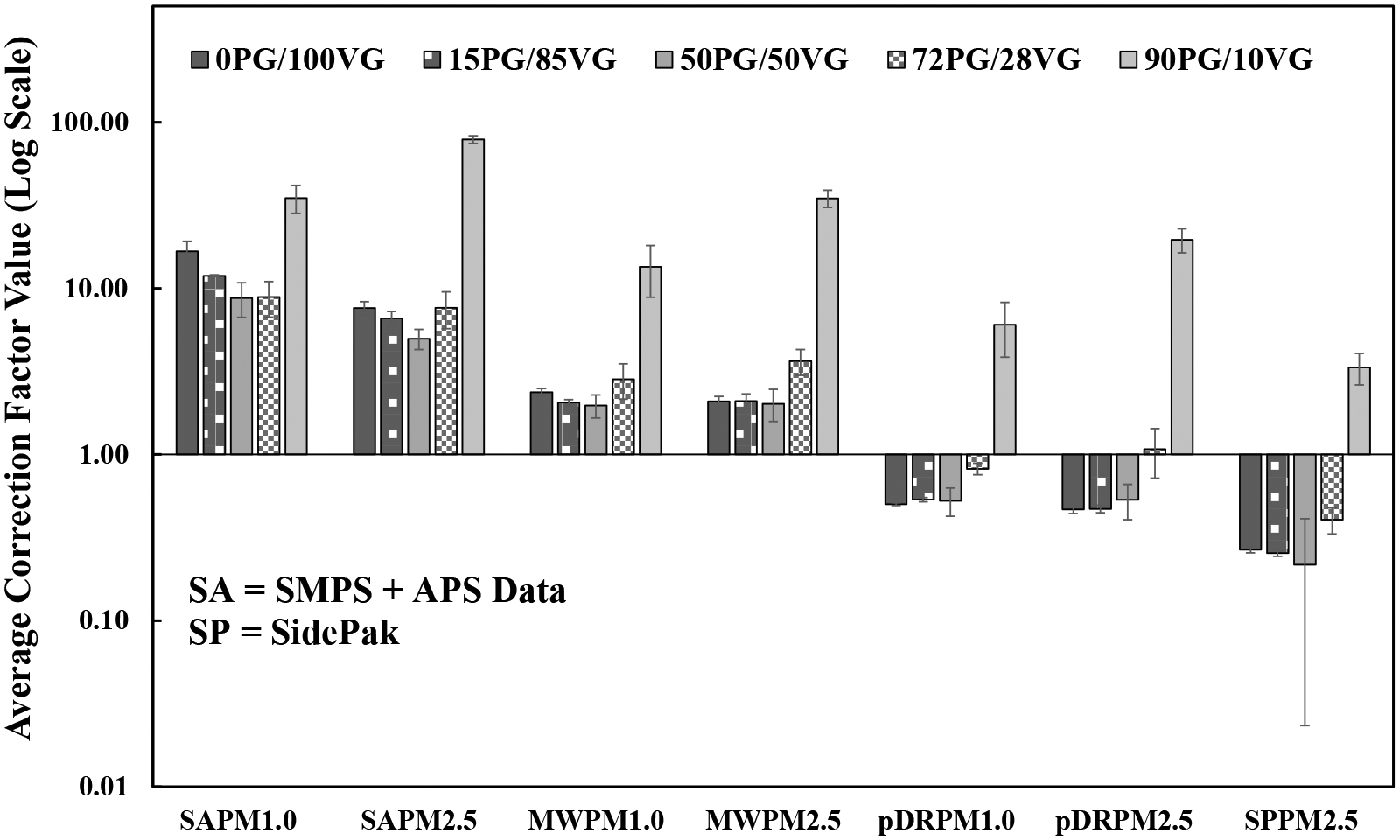
Correction factors for instruments of each particle size and each ECIG mixture ratio. Averages and standard deviation were based on three trials for each ratio.

The filter correction factor values for the SMPS + APS data, MiniWRAS, pDR, and SidePak are shown in Fig. 3. The y-axis was displayed in a log scale to distinguish between the low values in the figure. The y-axis error bars represent the standard deviation of the three trials. A correction factor of 1.0 indicates that the average real-time concentration collected from a monitor was equal to the discrete measurement collected on the filter. A value above 1.0 indicates that the monitor underestimated the discrete measurement, while a value below 1.0 indicates that the monitor overestimated the discrete measurement. The correction factors for all 5 ratios in all monitors differed and were either above or below 1.0. The 90PG/10VG ratio values were substantially high compared to the other ratios for the same monitor. Therefore, excluding 90PG/10VG ratio, for PM_2.5_, the correction factor range for each monitor was: SMPS + APS data (4.96–7.62), MiniWRAS (2.02–3.64), pDR (0.50–1.07), and SidePak (0.22–0.40). For PM_1_, the correction factor range for each monitor was: SMPS + APS data (4.96–16.69), MiniWRAS (2.02–2.83), and pDR (0.50–0.82).

The SMPS + APS data and the MiniWRAS were underestimated for all ratios. The pDR and SidePak were mostly overestimated, except for 72PG/28VG ratio for the pDR, with a value of 1.07 and the 90PG/10VG ratio for both monitors. The highest underestimation was found in 90PG/10VG ratio in the fitted PM_2.5_ SMPS + APS data, with a correction factor value of 79.00. The lowest correction factor was found in the SidePak in the 50PG/50VG ratio, with a value of 0.22. Therefore, indicating the highest underestimation and overestimation, respectively. The correction factor values for the SMPS + APS were similar ratios 1 through 4, except for PM_1_ SMPS + APS 0PG/100VG ratio was twice the value of the other ratios. For the MiniWRAS, pDR and SidePak, the correction factors were similar for ratios 1 through 3, and values increased for ratios 4 and 5.

The high PG values in 90PG/10VG ratio caused all the monitors to underestimate the filter values due to the high volatility of PG (David *et al*., 2020; Luo *et al*., 2021; Sousan *et al*., 2022b). The SMPS + APS correction factors were the highest, indicating a high underestimation. The SMPS and APS use sheath air so that the particle count does not exceed the maximum measurable number concentration. Therefore, the sheath air could contribute to the volatilization of the PG and VG liquid mixture, especially for the SMPS, with a sheath air to aerosol flow ratio of 6.6. The MiniWRAS underestimations could be explained by the device’s inability to handle high concentrations for particles smaller than 0.25 μm (Sousan *et al*., 2022a). The pDR and SidePak are photometers that are usually calibrated for dust aerosol. Therefore, the spherical nature of the liquid particles could generate high-intensity values causing overestimation compared to the filter values.

The correction factor values in this work were similar to Sousan *et al*. (2022a) for ratios 1 through 3 for the MiniWRAS, pDR and the SidePak. Sousan *et al*. (2022a) developed correction factors for the MiniWRAS, pDR, and SidePak for different ECIGs with known PG/VG mass ratios of 19PG/81VG, 27PG/73VG, and 35PG/65VG (Talih *et al*., 2020, 2021; VAPING, 2021). The correction factors were similar or slightly different for the pDR (0.45–0.68) and SidePak (0.13–0.20). For the MiniWRAS, the correction factors were also similar or slightly different for pod mods and disposable type ECIGs (0.64–2.66) and extensively different for Box Mod type ECIG (3.99–6.01). The similarities were likely due to similar ratios. When the liquids had high VG content, the correction factors were below unity for the pDR and SidePak, compared to unity and above unity with high PG content. In addition, the MiniWRAS correction factor values were similar at higher VG content, then correction factors increased with higher PG content. In addition, differences could be due to puffing regimen, device temperature control, wicking ability, and PG/VG ratios between the different ECIG types (Sousan *et al*., 2022b). Therefore, correction factors could be specific to the aerosol instrument, ECIG brand, and particle size.

### 3.3 Aerosol Size Distribution

**Fig. 4.**
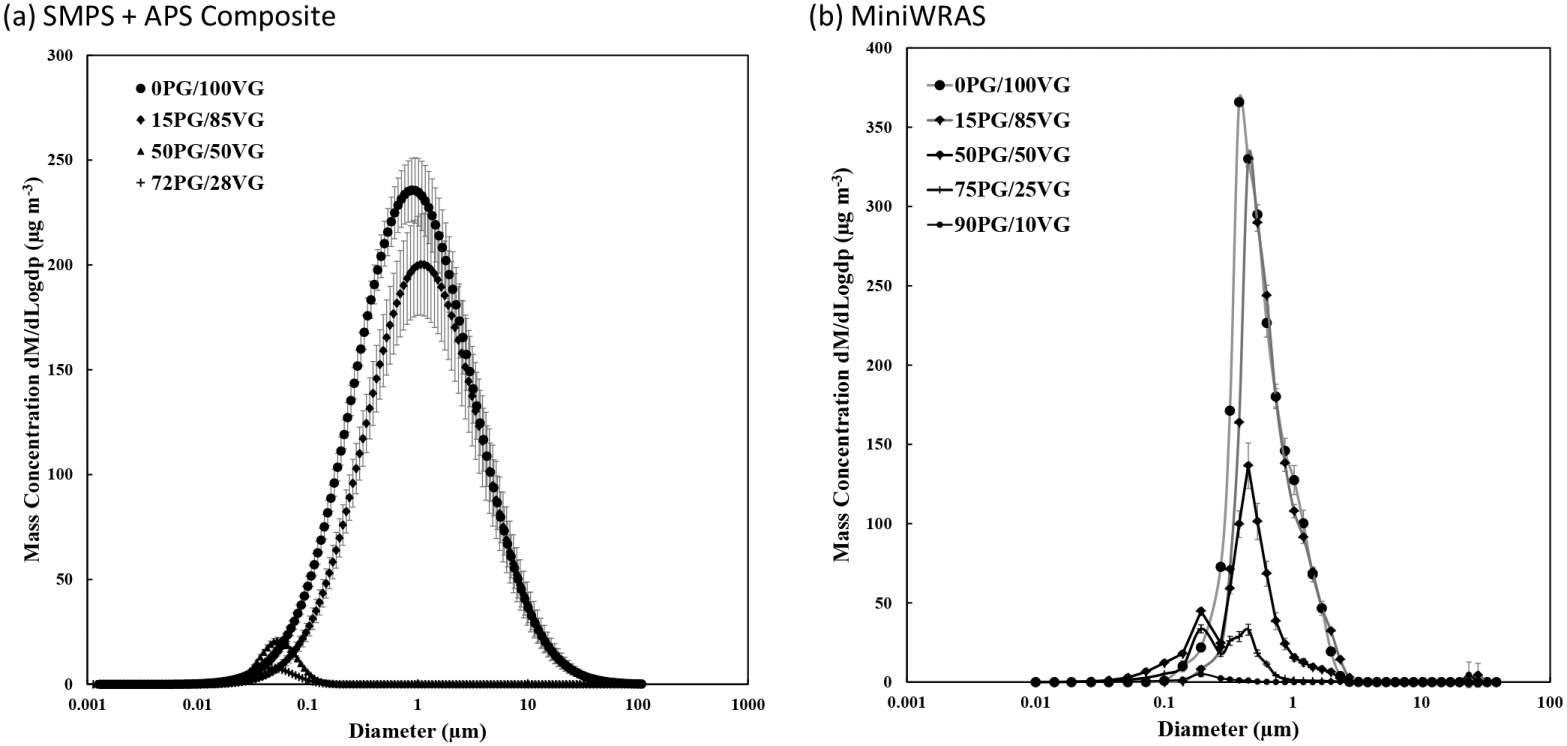
Aerosol size distribution by mass for (a) SMPS + APS fitted data and (b) MiniWRAS. X-axis represents the volumetric particle diameter. Each curve’s average and standard deviation were computed based on three measurements during a trial of each experiment. The Y-axis represents the standard deviation of the three measurements.

The MMD and GSD values for the SMPS, APS, SMPS + APS composite data, and the MiniWRAS are shown in Table 2. In addition, the aerosol size distribution by mass for the SMPS + APS fitted data, and MiniWRAS for different ratios are shown in Fig. 4. The TSI’s Data Merge Software was unable to calculate the SMPS + APS fitted data for the 90PG/10VG ratio due to the extremely high SMPS losses causing the concentration to be outside the software limits. The MMD range for each ratio was 0.04–0.40 for the SMPS, 1.33–3.81 for the APS, 0.05–0.76 for the SMPS + APS fitted data and 0.37–5.72 for the MiniWRAS. For all four ratios, the SMPS + APS fitted data were smaller than 1 μm. The combined SMPS + APS data were similar to the MiniWRAS values for the 0PG/100VG ratio (0.49 μm compared to 0.56 μm) and 15PG/85VG ratio (0.49 μm compared to 0.55 μm), respectively. However, 50PG/50VG ratio (0.05 μm compared to 0.43 μm) and 72PG/28VG ratio (0.76 μm compared to 0.37 μm), respectively, were significantly different. The SMPS losses due to PG volatilization can be observed in ratios 3 through 5 with an MMD value of 0.05 μm, compared to the APS and MiniWRAS, that both experienced losses resulting in MMD values of 3.81 and 5. 72 μm, respectively. Various fitting settings for scaling between the two devices were considered, and ultimately, the “Use Fit Estimate Method” provided the most appropriate fitting. The fitted SMPS + APS MMD values were smaller for most ratios compared to the MMD values measured by the MiniWRAS.

The differences between MMD and GSD values between the monitors are attributed to the differences between the sciences used for each monitor. In addition, the Data Merge Software uses a fitting model to calculate the MMD and GSD values. The SMPS + APS fitted data in Fig. 4 shows that both monitors contain different and higher bin sizes than the MiniWRAS. In addition to bin sizes, previous research has concluded that data merge software could cause discrepancies in particle size distribution compared to the MiniWRAS (Sousan *et al*., 2022a). Therefore, it is not surprising to see different values for these monitors and the combined SMPS + APS values.

The low concentrations for ratios 3, 4 and 5 may be explained by the large presence of PG with high volatilization and less particle loss (Li *et al*., 2021). The TSI’s Data Merge Software was unable to provide the aerosol size distribution for SMPS + APS fitted data for the 90PG/10VG ratio due to the low concentrations of the SMPS.

In comparison with this study, Sousan *et al*. (2022a) measured MMD using combined fitted data from the SMPS and optical particle counter (OPS), and the MiniWRAS for 5 different ECIG brands with known PG/VG ratios of 19/81, 27/73, and 35/65. The authors found that the MMD values were between 0.41–0.62 for the SMPS + APS fitted data and 0.37–0.50 for the MiniWRAS, which were similar for ratios 1 through 4.

Filter correction factors are important for real-time PM monitors, where instrument manufacturers are emphatic in using them to increase the accuracy of aerosol measurements (Sousan *et al*., 2022a). This study calculated the filter correction factors for PM concentrations from ECIG aerosol using different aerosol monitors in five different ECIG liquid mixtures. Correction factors were measured in an exposure chamber laboratory environment using a diaphragm pump to simulate real-life vaping behaviors. Filter correction factors determined in this study could be used to emulate real-life scenarios, where future studies should examine these values in real-life settings.

### 3.4 Study Limitations

The limitation of this study was using laboratory grade PG, VG, and nicotine liquids. Commercial grade liquids could introduce impurities that might provide different filter correction factors. In addition, the study did not add any flavorants to the PG/VG mixtures available when consumers purchase ECIG. Therefore, commercial PG, VG, nicotine liquids, and nicotine salts and flavorants could provide different results from this study. This study only focused on airborne PM and did not examine PM deposited on surfaces within the chamber, where future work may also examine surface depositions inside the chamber. Future work should focus on the effects of commercial-type products, nicotine salts and flavorants used to create in-home custom-made ECIG liquid mixtures on the correction factors developed for aerosol instruments. Future studies should also examine gas-phase chemicals that are generated from ECIG use in chamber settings. This study examined aerosol generated from one ECIG device type. While this approach was used to control for differences in aerosol generated due to between-device differences, future research may examine how devices of varying characteristics (e.g., device power) interact with PG/VG ratio.

## 4 CONCLUSIONS

This study shows that correction factors depend on the PG/VG ratios to some extent. Based on the PM_2.5_ measurement, the SMPS + APS data showed that correction factors are similar for most PG/VG ratios except those that have very high concentrations of PG, whereas the MiniWRAS, pDR, and SidePak correction factors were only similar for liquids with PG/VG ratios where there was at least 50% VG. Because commercially available liquids often vary in PG/VG ratios and some ECIG users make their own “do-it-yourself” liquids that also may vary in PG/VG ratio, it is important to understand the effects of different PG and VG mixtures on the aerosol generated and the correction factors determined for different aerosol instruments. In terms of raw aerosol generated, this study confirmed that higher levels of VG in an ECIG ratio are associated with higher concentration due to low VG volatility. Field studies need to evaluate using correction factors developed in laboratory settings for aerosol instruments based on the ECIG brand used by consumers or the custom-made liquids for nicotine delivery.

## Supplementary Material

Supplemental Information

## Figures and Tables

**Table 1.** Compositions of each ECIG liquid ratio based on the preparade mixture and the chemical analysis of the sample. For each ration, a 5% nicotine by weight was added to each ratio.

Ratio Mixture (M)/Chemical Analysis (CA)	Propylene Glycol	Vegetable Glycerin	Assumed Density (mg mL^−1^)
0PG/100VG (M)	0%	100%	1260
25PG/75VG (M)	25%	75%	1205
*15PG/85VG* (*CA*)			
50PG/50VG* (M)	50%	50%	1150
75PG/25VG (M)	75%	25%	1095
*72PG/28VG* (*CA*)			
90PG/10VG (M)	90%	10%	1062
*90PG/10VG* (*CA*)			

* Chemical analysis was not performed on the mixture after the experiment due to insufficient remaining liquid.

**Table 2.** The MMD and GSD were calculated for the SMPS, APS, SMPS + APS fitted data, and MiniWRAS on 3 different measurements for each device. The MMD standard deviation is showed in-between brackets.

Ratio (PG/VG)	SMPS		APS		SMPS + APS (UFEM)		MiniWRAS
	MMD (μm)	GSD	MMD (μm)	GSD	MMD (μm)	GSD	MMD (μm)
0PG/100VG	0.32 [0.03]	1.9	1.79 [0.01]	2.2	0.49 [0.24]	2.8	0.56 [0.02]
15PG/85VG	0.40 [0.02]	2.0	1.73 [0.01]	2.25	0.49 [0.25]	3.3	0.55 [0.03]
50PG/50VG	0.05 [0.03]	1.8	1.33 [0.77]	2.39	0.05 [0.03]	1.6	0.43 [0.06]
72PG/28VG	0.05 [0.01]	2.6	1.41 [0.09]	2.5	0.76 [1.23]	1.9	0.37 [0.00]
90PG/10VG	0.04 [0.0]	2.0	3.81 [1.38]	2.8	NA	1.6	5.72 [2.82]

UFEM = Use Fit Estimate Method.
