# Supplementary material for: Effects of E-Cigarette Liquid Ratios on the Gravimetric Filter Correction Factors and Real-Time Measurements
Source: Aerosol Air Qual Res. Author manuscript; Available in PMC 2024 Mar 18. (PMC10947168; doi:10.4209/aaqr.230011)
Supplement: Supplemental Information [file NIHMS1929730-supplement-Supplemental_Information.docx]

| **Table S1: The average PM_1_ and PM_2.5_ concentrations (µg m^-3^) of each ratio for the SMPS+APS, MiniWRAS, pDR, and SidePak.** | | | | | |
| --- | --- | --- | --- | --- | --- |
| **PG/VG Ratio** | **0PG/100VG** | **15PG/85VG** | **50PG/50VG** | **72PG/28VG** | **90PG/10VG** |
| ***PM_1_*** |  |  |  |  |  |
| **SMPS+APS** | 303 | 345 | 161 | 67 | 5 |
| **MiniWRAS** | 2,113 | 2,068 | 712 | 210 | 14 |
| **pDR** | 9,968 | 7,560 | 2,766 | 746 | 20 |
|  |  |  |  |  |  |
| ***PM_2.5_*** |  |  |  |  |  |
| **SMPS+APS** | 708 | 726 | 292 | 104 | 6 |
| **MiniWRAS** | 2,524 | 2,400 | 745 | 215 | 16 |
| **pDR** | 11,262 | 10,153 | 2,846 | 785 | 28 |
| **SidePak** | 19,622 | 18,722 | 6,794 | 1,964 | 174 |
|  |  |  |  |  |  |

**Propylene Glycol and Vegetable Glycerin ratio chemical analysis method**

**Materials**

High purity propylene glycol (TCI, >99.0%, CAS RN 57-55-6) and vegetable glycerin (J.T. Baker, USP grade, CAS RN 56-81-5) were used for preparation of standard solutions and control samples. Triethylene glycol (TEG) (Thermo Scientific, >99%, CAS RN 112-27-6) was used as an internal standard at 20 µg/mL. N,O-Bis(trimethylsilyl)-trifluoroacetamide (BSTFA) (TCI, >95.0%, CAS RN 25561-30-2) was used for derivatization prior to analysis by gas chromatography with flame-ionization detection (GC-FID). The diluting solvent for all solutions was high-purity acetonitrile (Burdick and Jackson, LCMS grade, CAS RN 75-05-8).

**PG/VG Quantitation**

The method used for quantification of PG and VG was derived from the procedure described by EL-Hellani et al. (2016) with modifications. The most significant changes include use of acetonitrile as the diluting solvent, triethylene glycol as the internal standard, sample aliquots by weight, and analysis by GC-FID.

Stock sample solutions were prepared by dissolving approximately 40 mg ECIG liquid (accurately weighed) in 50 mL of acetonitrile. Fifty microliters (50 µL) of stock sample was added to an amber glass 2-mL autosampler vial containing 500 µL BSTFA, 200 µL TEG in acetonitrile, and 250 µL acetonitrile for a final volume of 1 mL and tightly closed with a screw-cap prior to derivatization at 70°C for 30 minutes.

Standard preparations were prepared by dilution of a stock solution containing PG and VG in acetonitrile. A series of working standard solutions (5-50 µg/mL) were prepared by transferring increasing volumes of stock standard solution to 2-mL autosampler vials containing the specified amounts of BSTFA and TEG with decreasing volumes of acetonitrile to obtain a final volume of 1 mL each.

A laboratory control sample was prepared by combination of accurately weighed 1-g aliquots of PG and VG in a 20-mL amber glass bottle using gentle heat and mixing. The working standard and working control solutions were prepared and derivatized alongside the working sample preparations. The 50/50% w/w control sample was estimated to contain 48/52% w/w PG/VG against the quantitation standards.

**GCFID conditions**

Analysis was performed on an Agilent 6890N gas chromatograph with flame-ionization detector and Agilent HP-5 column (30 m x 0.32 mm, 0.25 µm film) using 1 µL injection volume in splitless mode. The helium carrier gas flowrate was 4.1 mL/min. The inlet and detector temperature were 250°C. The initial oven temperature was held at 60°C for 3 minutes. The oven was then ramped at 10°C/min to 80°C, ramped at 30°C/min to 120°C, held for 1 minute, ramped at 10°C/min to 180°C, then finally ramped at 30°C/min to 230°C and held for 1 minute. Nitrogen, air, and hydrogen detector gases were 20, 400, and 30 mL/min, respectively.

**References**

EL-Hellani, A., Salman, R., El-Hage, R., Talih, S., Malek, N., Baalbaki, R., Karaoghlanian, N., Nakkash, R., Shihadeh, A., Saliba, N.A. (2016). Nicotine and Carbonyl Emissions From Popular Electronic Cigarette Products: Correlation to Liquid Composition and Design Characteristics. Nicotine & Tobacco Research 20, 215-223. <https://doi.org/10.1093/ntr/ntw280>
